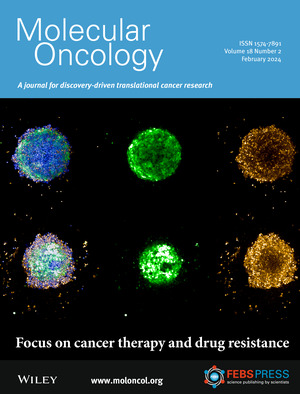# Issue Information

**DOI:** 10.1002/1878-0261.13456

**Published:** 2024-02-07

## Abstract

The issue features content focused on fighting cancer by various therapeutic approaches and overcoming drug resistance. Read the Viewpoint on drug resistance by Mariangela Russo in pp. 241–244 and the meeting report on strategies to decrease inequalities in cancer therapeutics, care and prevention by Ulrik Ringborg *et al*. in pp. 245–279.

On the cover: mPGES‐1 inhibition enhances the efficacy of vinblastine in neuroblastoma spheroids, illustrated with live/dead probes. Read the full article by Ahlem Zaghmi *et al*. in pp. 317–335.